# Aviation effects on already-existing cirrus clouds

**DOI:** 10.1038/ncomms12016

**Published:** 2016-06-21

**Authors:** Matthias Tesche, Peggy Achtert, Paul Glantz, Kevin J. Noone

**Affiliations:** 1 Department of Environmental Science and Analytical Chemistry (ACES), Stockholm University, SE-10691 Stockholm, Sweden

## Abstract

Determining the effects of the formation of contrails within natural cirrus clouds has proven to be challenging. Quantifying any such effects is necessary if we are to properly account for the influence of aviation on climate. Here we quantify the effect of aircraft on the optical thickness of already-existing cirrus clouds by matching actual aircraft flight tracks to satellite lidar measurements. We show that there is a systematic, statistically significant increase in normalized cirrus cloud optical thickness inside mid-latitude flight tracks compared with adjacent areas immediately outside the tracks.

## Abstract (web summary)

The extent to which air traffic affects the optical thickness of existing cirrus clouds in the upper atmosphere remains unknown. Here, the authors compare cirrus cloud properties inside and outside of real flight tracks and show that optical thickness is significantly increased within the flight path.

## Graphical abstract

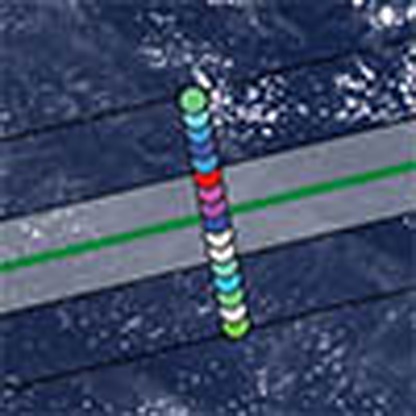

Air traffic is known to have an immediate and noticeable effect on clouds in the upper troposphere. New clouds that form due to aircraft effluent are called contrails^1^,^2^, and may develop into more persistent and widespread contrail cirrus. Boucher^3^ was the first to realize that aviation might have a strong influence on the occurrence rate of cirrus clouds. Previous studies of contrail optical properties are either based on passive remote sensing in which contrails are identified as linear features in scenes of brightness temperature differences^4^,^5^,^6^ or modelling studies in which contrails are formed when favourable meteorological conditions are reached^7^. The life cycle of contrails and aviation-induced cirrus, their radiative forcing and feedback on natural clouds have been studied by treating them as an independent cloud class in a climate model^8^. The study by Iwabuchi *et al*.^9^ is the only one so far that has used height-resolved observations from space-borne lidar measurements to investigate the physical and optical properties of contrails. In their approach, the authors used passive MODIS (moderate resolution imaging spectroradiometer) observations to identify contrails for a subsequent detailed analysis of CALIOP (cloud-aerosol lidar with orthogonal polarization) observations.

In general, aviation-induced clouds (that is, contrails and contrail cirrus) have been found to be optically thin^10^,^11^, and their climatic effects have been estimated to be minor^4^,^12^,^13^,^14^,^15^ even when considering their entire life cycle^8^,^16^. The effect of contrails embedded in natural cirrus is a mechanism that currently has neither been studied nor assessed for its radiative effect on climate^8^,^15^,^16^,^17^.

While optically thick cirrus clouds have a net cooling effect on surface temperature, optically thin cirrus clouds, like greenhouse gases, can have a warming effect^15^,^18^. Aircraft emissions and contrails at cirrus altitudes have the potential to either cause optically thin cirrus clouds to form (that would have a warming effect on surface temperatures) or increase the optical thickness of existing clouds (or induce new optically thick clouds), thus, causing a net cooling effect. Enhanced observations of the effects of aircraft on cirrus cloud properties are needed to help bound and quantify these possible effects.

The aim of this study is to test the hypothesis that contrails formed within natural cirrus clouds have no measurable immediate effect on cirrus optical depth inside and outside flight tracks in the upper troposphere. We combine data of aircraft flight tracks with spaceborne lidar observations to investigate the effect of aviation on the optical thickness of already-existing cirrus clouds. We detect a statistically significant 22% increase in normalized cirrus optical thickness in mid-latitude air traffic flight tracks compared with adjacent areas outside the flight tracks.

## Results

### Data sources

We have used commercially available flight track data from FlightAware.com for aircraft serving the major connections between the west coast of the United States and Hawaii in the years 2010 and 2011. Actual flight data (measurements from the aircraft) are received from Air Navigation Service Providers and Automatic Dependent Surveillance—Broadcast receivers. In intervals when no data is received from the aircraft itself, positions are interpolated between the last two reported positions. We consider commercial airline connections between Seattle (KSEA), San Francisco (KSFO), Los Angeles (KLAX) and Honolulu (PHNL).

We derive information about cirrus optical thickness (COT), cloud base and top height, cloud geometrical depth (vertical extent) and mean extinction coefficient from observations with the CALIOP instrument aboard the cloud-aerosol lidar and infrared pathfinder satellite observations (CALIPSO) satellite^19^. Details on the selection of CALIPSO data used in this study are given in the Methods section.

### Approach

**Figure 1:**
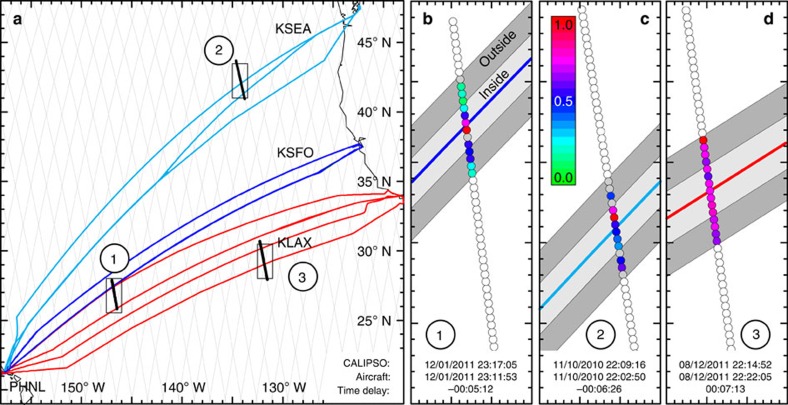
Overview of the analysis approach. (**a**) Typical aircraft flight tracks (coloured lines) and CALIPSO satellite trajectories (grey lines for 16-day cycle, black lines for example cases). (**b**–**d**) Close-up of three example overpasses indicated in **a** with values of normalized cirrus optical thickness (coloured dots) and illustration of the inner and outer track (light and dark grey shading, respectively). White and grey dots in **b**–**d** refer to data that have not been considered in the analysis and do not fulfil the quality assurance criteria, respectively. Times of CALIPSO and aircraft overpasses are given at the bottom of **b**–**d**. Negative and positive time delay values indicate that the aircraft arrived at the scene before and after, respectively, the satellite overpass.

Figure 1 illustrates our approach. Typical flight tracks for connections between Seattle (KSEA), San Francisco (KSFO), Los Angeles (KLAX) and Honolulu (PHNL) are shown as thick coloured lines. CALIPSO orbits are indicated as thin grey lines in the figure. The inset shows normalized COT (nCOT; see below) at 532 nm across each of the flight corridors between Los Angeles, San Francisco, Seattle and Honolulu. In cases 1 and 2 aircraft had passed the area <30 min before the CALIPSO overpass. In case 3, CALIOP observed the location of the flight track before the passage of the aircraft. For these cases cirrus clouds were present at the flight level of the aircraft. For the cases 1 and 2 where the aircraft arrived before the satellite overpass, CALIOP nCOT was clearly larger for the inner part of the flight track compared with clouds present on either side—creating a ‘plane track' signature caused by an embedded contrail or another effect on the cloud caused by the aircraft.

### Categories for data analysis

**Figure 2:**
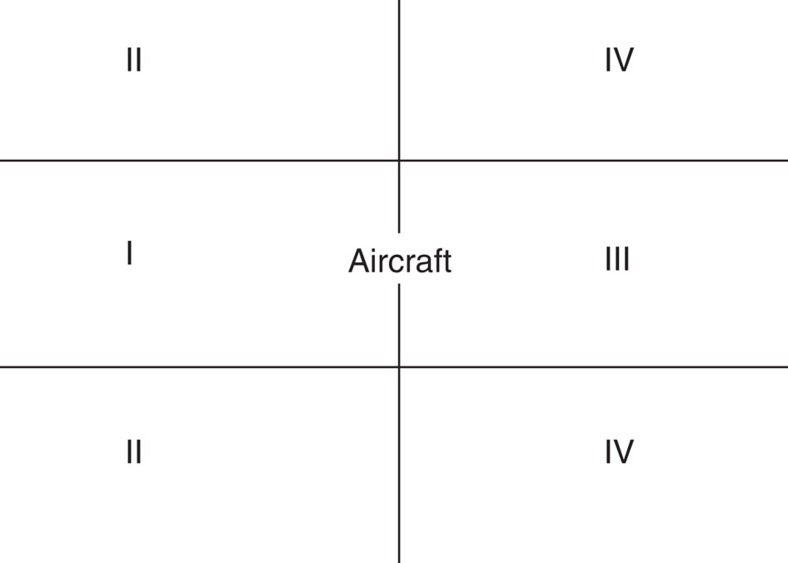
Categories for data analysis. Category I: inside the flight track, ahead of the aircraft. Category II: outside the flight track, ahead of the aircraft. Category III: inside the flight track, behind the aircraft. Category IV: outside the flight track, behind the aircraft.

We accumulated data for these three air traffic corridors during 2010 and 2011 in which the absolute difference between aircraft and CALIPSO arrival times was 30 min or less. We classified the data into four categories illustrated in Fig. 2: (I) inside flight track, ahead of aircraft; (II) outside flight track, ahead of aircraft; (III) inside flight track, behind aircraft; and (IV) outside flight track, behind aircraft.

If our hypothesis that aircraft have no observable effect on cirrus cloud properties is true, then there should be no statistically significant differences in COT or in nCOT between the four categories shown in Fig. 2. If the hypothesis is false, and aircraft emissions do have an impact on cloud properties, clouds in category III should have different nCOT compared with the other categories.

### Normalization of cirrus cloud optical thickness

**Figure 3:**
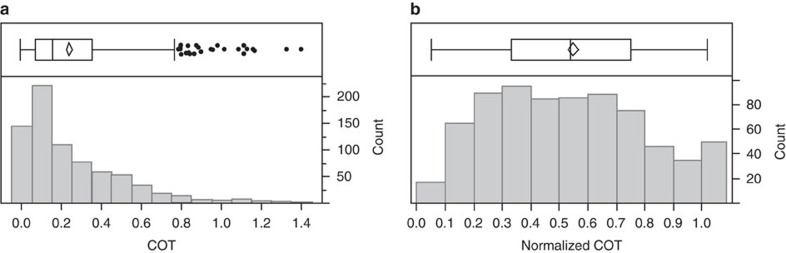
Frequency distribution of cirrus optical thickness. Distribution of absolute COT (**a**) and nCOT (**b**) for cases where the maximum cloud layer depth is 2.5 km. The data come from all four categories. The box-and-whisker plots show the 25 and 75% quartiles as the box, with the median value being the vertical line inside the box and outliers shown as dots. The diamond inside the box indicates the mean value (vertical vertices) and ±95% confidence interval (horizontal vertices) for the data. The number of observations is shown on the right vertical axis. Note that the highest bin in **b** only contains values of 1.0.

As a result of natural variability in the COT of unperturbed cirrus clouds, our COT data set is skewed towards large values. The histograms in Fig. 3 show the distribution of 720 5-km CALIOP data points with a maximum cloud geometrical depth of 2.5 km. The distribution of absolute COT in Fig. 3a has a skewness of 1.57. We normalize COT with respect to the maximum CALIOP value for the overpass. Figure 3b shows the frequency distribution for the same data after normalization. The mean and median values are now essentially equal, and the distribution has a skewness value of 0.18. The distributions of COT and nCOT for cases with maximum cirrus geometrical depths different from 2.5 km have very similar shape and skewness (not shown). The rightmost column in Fig. 3b bears further explanation. nCOT values in these data vary from a minimum of 0.03 to a maximum of 1. The column limits in Fig. 3b go from 0 to 0.0999 for the first column, 0.1 to 0.1999 for the second and 0.9 to 0.9999 for the next-to-last column. Since the true COT for each of the overpasses we accumulated will have at least one maximum value, each pass for nCOT will have at least one value of unity. The rightmost column shows these unity values, which are too large to be included in the interval from 0.9 to 0.9999. The rightmost column should not be interpreted as values larger than one, but rather values of identically one.

The normalization ensures that the data are more normally distributed (a requirement for the statistical tests we use), and the analysis is not biased by a few large values. If our hypothesis that aircraft have no measurable effect on cirrus cloud optical properties is true, there should be no statistically significant differences in mean COT and mean nCOT between these four categories. Otherwise, the mean values for category III should be different than for the other categories, which should not exhibit any differences in mean values.

### Effects of advection and other aircraft

**Figure 4:**
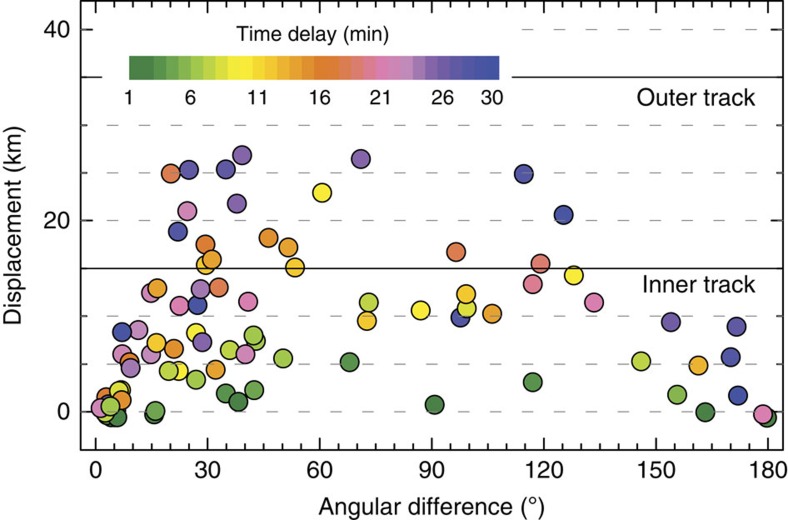
Effect of advection. The effect of advection presented as displacement perpendicular to the flight track versus the angular difference between aircraft heading and wind direction. The colour coding refers to the absolute time difference between aircraft and CALIPSO.

We use ERA-Interim wind speed *v* and direction in combination with speed and heading of the aircraft at the location and height of the crossing between flight track and CALIPSO overpass to account for the effect of advection. The displacement *D* perpendicular to the flight track is derived as *D=*Δ*t v* sin Δdir, where Δdir is the angular difference between aircraft heading and wind direction and Δ*t* is the absolute value of the time delay between aircraft and CALIPSO overpass (Fig. 4). We then omit cases that are associated with a displacement larger than 30 km perpendicular to the flight track. We cannot simply calculate how far the emissions from the aircraft would be moved in the time interval between the satellite overpass and the aircraft passage given a constant wind and ‘move' our observations there. We only have observations along the line of the CALIPSO orbit, as illustrated in panels b–d in Fig. 1 and in Supplementary Fig. 3. Let us choose the CALIPSO/flight track crossing point as the origin for our coordinate system. A hypothetical wind vector is shown as a thick purple arrow in Supplementary Fig. 3. Given this wind vector, material emitted by the aircraft at the crossing point (1) would be advected to point (2) in the time interval between the passage of the aircraft and the time that CALIPSO observes this point. Point (2) is not on the CALIPSO track. To perform this sort of advection calculation properly for this system, we would need to first calculate the wind vector component along the CALIPSO track to find point (3), then use the wind vector to calculate the origin of the air that CALIPSO would have observed at this new point (which is point (4) in the illustration). Given the CALIPSO/flight track geometry, the lag time between the satellite and aircraft passing point (1) and the wind vector, this new point of origin may or may not be on the flight track aft of the aircraft. In any case, this iterative calculation would be necessary for each crossing.

In addition, winds in the atmosphere are not constant. Turbulence (even for a constant wind speed) can redistribute emissions around the centreline calculated in the manner described above, and uncertainties in the ERA-Interim winds also need to be taken into account. To use this approach we would at a minimum need to perform Gaussian plume dispersion calculations for a line source, and augment these with estimates of the effects of uncertainties in the ERA-Interim wind fields. This plume advection approach is illustrated as a diffuse horizontal line in Supplementary Fig. 3. The result of these calculations would be a flight corridor with a new location, centred around a particular line, but with a probability distribution of location.

Figure 4 shows how far advection would have moved any emissions from the aircraft perpendicular to the flight track in the time interval between the passage of the aircraft and the satellite overpass. In the majority of cases, advection does not move material outside what we define as the inner flight track, and therefore a more sophisticated calculation is not needed in this approach.

To account for the effect of other aircraft on the same flight track we omit cases in which the delay between any previous aircraft and our flight of interest was <30 min. Supplementary Fig. 4 illustrates how advection may influence the properties of the air in the categories we use for analysis. Further details on the effect of advection on our findings are provided in the Supplementary Discussion, as well as in Supplementary Tables 1 and 2.

### Findings for clouds with different geometrical thickness

**Figure 5:**
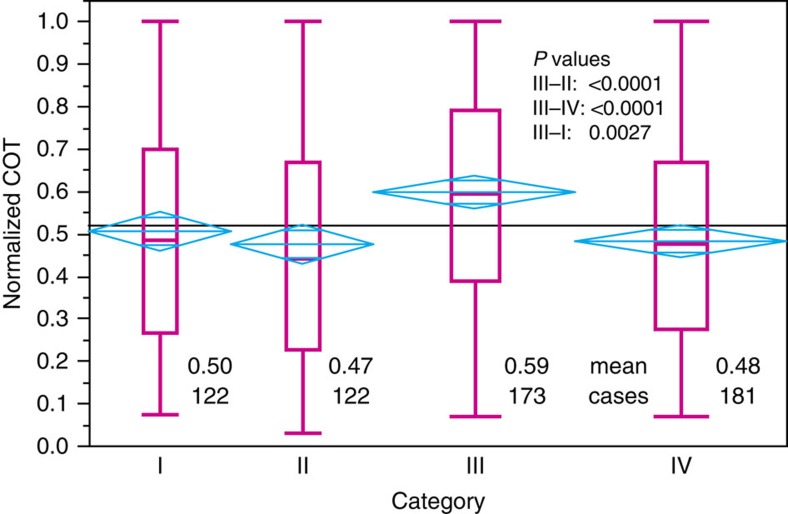
Cirrus cloud optical thickness per category for a maximum cirrus geometrical depth of 2.5 km. The magenta box-and-whisker plots show the quantiles for the data in each category from a one-way analysis of variance using the JMP software package. Mean diamonds (cyan) indicate the 95% confidence intervals for the mean values of each of the categories. If the upper and lower horizontal lines overlap, there is no statistically significant difference in means. Numbers in the lower part of the figure give the mean value and number of observations (that is, CALIPSO L2 5-km points) in each category. The horizontal grey line represents the overall mean value.

The results of this analysis are shown in Fig. 5. For brevity we present the results for cases in which the maximum cirrus depth was 2.5 km. The mean nCOT for category III (0.59) is significantly higher than for the other three categories (III−II: *P*<0.0001; III−IV: *P*<0.0001; III−I: *P*=0.0027). In terms of true COT, the category 3 mean value was 0.30, while the means of the other categories were as follows: I, 0.27; II, 0.26; IV, 0.26. Thus, the mean COT for category III was 14% higher than the other categories, though statistically significant only at the 93% confidence level due to the skewness of the data. Differences between the other categories were not statistically significant. We examined cases for different maximum cloud layer depths (Supplementary Fig. 1). Table 1 reveals that the difference in nCOT for clouds inside and outside the flight track aft of the aircraft persists for all cases of maximum cirrus geometrical depth. The difference between category III and the other categories ahead of the aircraft is significant only for geometrically thin clouds.

**Figure 6:**
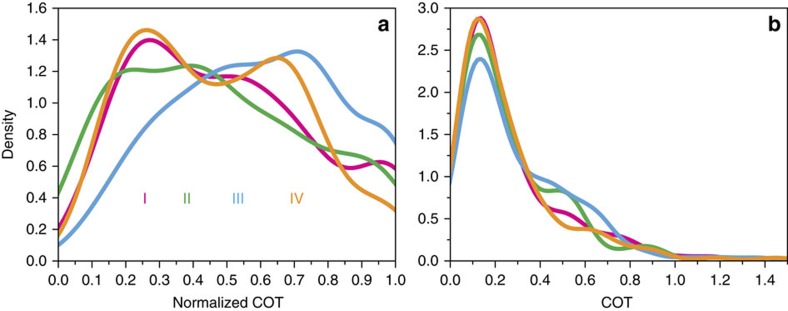
Distribution of cirrus optical thickness per category. Frequency of observation of nCOT (**a**) and absolute COT (**b**) for all cases, with maximum cirrus geometrical depth of 2.5 km (that is, the data presented in Fig. 2). Colours refer to the different categories. Category I (magenta): inside the flight track, ahead of the aircraft. Category II (green): outside the flight track, ahead of the aircraft. Category III (blue): inside the flight track, behind the aircraft. Category IV (orange): outside the flight track, behind the aircraft.

Figure 6 gives the frequency of observation of both COT and nCOT for the case of 2.5 km maximum cirrus geometrical depth. nCOT in category III shows fewer low values and a larger proportion of high values than the other categories. This difference in the frequency distributions for the different categories decreases for increasing maximum cirrus geometrical depth (not shown). The distributions become essentially indistinguishable from each other by a maximum cirrus geometrical depth of 5.0 km.

We have falsified our hypothesis, and observed a detectable 22% increase in nCOT in flight tracks for cases where the aircraft was 30 min or less ahead of the satellite overpass. We examined our data for differences in COT between day- and night-time CALIPSO overpasses. We did not observe any significant day–night differences in nCOT, and therefore have included both kinds of observations in our analysis (Supplementary Fig. 2).

## Discussion

Air traffic corridors are far more prevalent in the northern hemisphere than in the southern hemisphere, so we anticipate any climatic effects these embedded contrails may have will be more pronounced there. Even though cloudiness may already be changed by earlier aircraft, we can isolate the effect of a single aircraft on cloud properties. Since the effect of aircraft on cloud properties may well last longer than 30 min the overall effect of air traffic on cloud properties may be larger than the values estimated here. Estimating the climatic effects of embedded contrails is beyond the scope of this paper; however, given the broad coverage of air traffic corridors in the northern hemisphere, embedded contrails as identified in this study are potentially an important and not yet considered contributor to the non-CO_2_ effects of aviation on climate^17^.

Further work will be needed to quantify the effect identified in this study. Initially, detailed radiative transfer modelling is needed to assess the impact of an increase in COT on the Earth's radiative budget. From the modelling perspective, future studies will need to estimate the magnitude of the observed effect on a global scale and assess its contribution to the overall non-CO_2_ effects of aviation on climate. The increase in cirrus optical depth may result from the emitted soot in the first few seconds within the plume. Soot particles are not efficient ice nuclei. They rather form droplets when water saturation is reached in the plume and freeze subsequently^20^. Hence, the effect on the microphysics of the cirrus is an open question, and will require detailed microphysical modelling to address.

## Methods

### Overall approach

Our empirical approach is based on the ship tracks methodology^21^. Ship tracks occur in marine stratocumulus clouds in locations where cloud albedo increases due to particulate emissions from ships^22^,^23^. Here we apply a similar observational approach—we investigate the optical and geometrical properties of cirrus clouds within known flight tracks (that is, a region affected by aircraft exhaust and contrails) to the ones immediately adjacent on either side of the flight track (that is, the unperturbed region outside of the main flight routes). To apply this approach we first need to identify regions in which we are likely to encounter cirrus clouds that are influenced by air traffic. Favourable regions for this study should fulfil the following criteria: the aircraft should be flying below the tropopause and at cirrus level, tropical convection should be of minor influence on cloud formation (that is, the region should lie outside the ITCZ band), and the area should be a sufficiently large so that CALIPSO will have multiple transects in a reasonable amount of time. These criteria rule out the intercontinental polar routes, since in these regions the aircraft are often flying above the tropopause level. The most promising routes are in the northern hemisphere mid-latitudes. Data on aircraft location are used to identify flight tracks.

### Spaceborne lidar observations

CALIOP is an elastic-backscatter lidar that emits linearly polarized laser light at 532 and 1,064 nm and features three measurement channels. The CALIPSO lidar has been operational since June 2006. An overview of the instrument and retrieval algorithms can be found in Winker *et al*.^19^. CALIPSO is a polar orbiting satellite with a return cycle of 16 days.

The spatial and optical properties of cirrus clouds as derived from CALIOP observations have been carefully validated with airborne lidar measurements^24^,^25^,^26^,^27^. CALIOP observations became an invaluable tool for regional and global studies on the occurrence and properties of cirrus^28^,^29^,^30^ and sub-visual cirrus^31^,^32^. CALIPSO's location in the A-Train satellite constellation furthermore enables the addition of further co-located measurements to the lidar data for advanced cirrus studies^29^,^32^.

CALIOP observations in the area 15–55° N and 115–155° W for the years 2010 and 2011 have been considered in this study. We use the level 2 version 3.01 (January 2010–October 2011) and 3.02 (November 2011 to December 2011) 5-km Cloud Layer products. Data are provided with a horizontal resolution of 5 km and refer to layers starting with the uppermost detected feature.

### CALIOP data quality assurance

We only considered CALIOP observations for which cirrus (feature type=cloud, sub-type=cirrus^33^, uppermost two layers are considered) occurs at the altitude of a coinciding flight track. For quality assurance we also require these features to be detected at 5-km averaging intervals (Horizontal_Averaging=5) and to show an Extinction_QC_Flag_532 of zero^34^. Note that data points with an Extinction_QC_Flag_532 other than zero are virtually absent in our data set. A detailed description of the CALIPSO products can be found in the CALIPSO's Data User Guide^35^.

### CALIOP data selection

We only consider the seven 5-km CALIOP data points to the north and south of the flight track, respectively. In addition, we require that at least half of these points (that is, 7 out of 14) show valid COT that fulfils our quality assurance criteria. This ensures that we observe a signal corresponding to homogeneous cirrus rather than natural variability of cirrus optical properties. We consider the three closest CALIPSO observations on either side of the aircraft track to form the inner part of the flight track (Fig. 1). Depending on the angle between the flight track and CALIPSO ground path, the inner part of the flight track spreads over up to 15 km to the north and south of the aircraft track. This distance agrees with the typical contrail width of about 5 km^9^ and allows compensating for minor advection effects caused by the 30-min (maximum) time interval between the aircraft and CALIPSO overpasses. Points (4)–(7) in Fig. 1b–d represent the unperturbed conditions outside of the flight track. This approach provides us with an overall number of data points that is balanced with respect to the inner and outer parts of the flight track.

### Difference between CALIOP night-time and day-time observations

Owing to the influence of daylight noise CALIOP feature detection sensitivity is higher at night than during day time. We therefore examined the data for differences in day- and night-time measurements. Examples of the mean nCOT for cases in which the maximum cirrus geometrical depth was 2.5 and 5.0 km are shown in Supplementary Fig. 2. We did not observe any significant day–night differences in normalized COT, and therefore have included both kinds of observations in our analysis.

### Data availability

The data that support the findings of this study are available on request from the corresponding author (K.J.N.).

## Additional information

**How to cite this article**: Tesche, M. *et al*. Aviation effects on already-existing cirrus clouds. *Nat. Commun.* 7:12016 doi: 10.1038/ncomms12016 (2016).

## Supplementary Material

Supplementary Information Supplementary Figures 1-4, Supplementary Tables 1-2 and Supplementary Discussion.

## Figures and Tables

**Table 1:** Statistical significance between all categories pairs with *P*≤0.05, subdivided with respect to maximum cirrus geometrical depth.

Maximum cirrus geometrical depth (km)	Significant differences between categories
2.0	III−IV, III−II, III−I
2.5	III−IV, III−II, III−I
3.0	III−IV, III−II
4.0	III−IV
5.0	III−IV
6.0	III−IV

The tests were performed for all category pairs.
