# Supplementary material for: Aviation effects on already-existing cirrus clouds
Source: Nat Commun. 2016 Jun 21;7:12016. doi: 10.1038/ncomms12016 (PMC4919534; doi:10.1038/ncomms12016)
Supplement: Supplementary Information — Supplementary Figures 1-4, Supplementary Tables 1-2 and Supplementary Discussion. [file ncomms12016-s1.pdf]

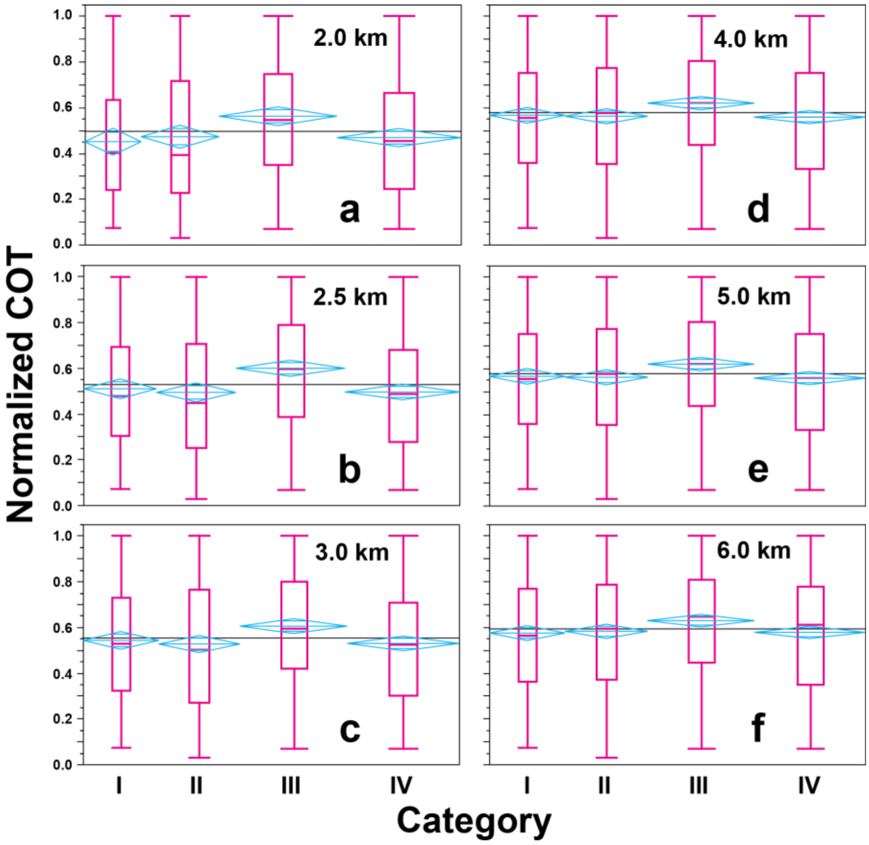

Supplementary Figure 1 | Effect of cloud thickness. Normalized cirrus optical thickness (COT) for categories I-IV with respect to maximum cirrus geometrical depth. The magenta box-and-whisker plots show the quantiles for the data in each category from a one-way analysis of variance using the JMP software package. Mean diamonds (cyan) indicate the 95% confidence intervals for the mean values of each of the categories. If the upper and lower horizontal lines overlap, there is no statistically significant difference in means. The horizontal grey line represents the overall mean value.

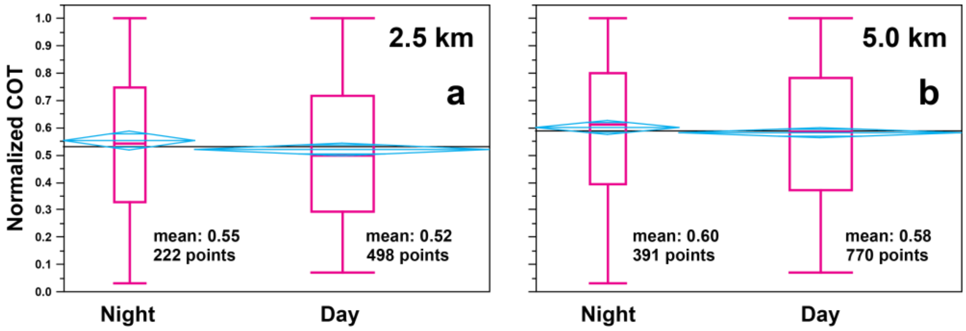

Supplementary Figure 2 | Investigation of daytime measurement noise. Mean normalized cirrus optical thickness (COT) for night and day Cloud-Aerosol Lidar and Infrared Pathfinder Satellite Observations (CALIPSO) satellite overpasses and maximum cirrus geometrical depth of 2.5 km (a) and 5.0 km (b). The magenta box-and-whisker plots show the quantiles for the data in each category from a one-way analysis of variance using the JMP software package. Mean diamonds (cyan) indicate the 95% confidence intervals for the mean values of each of the categories. If the upper and lower horizontal lines overlap, there is no statistically significant difference in means. The horizontal grey line represents the overall mean value.

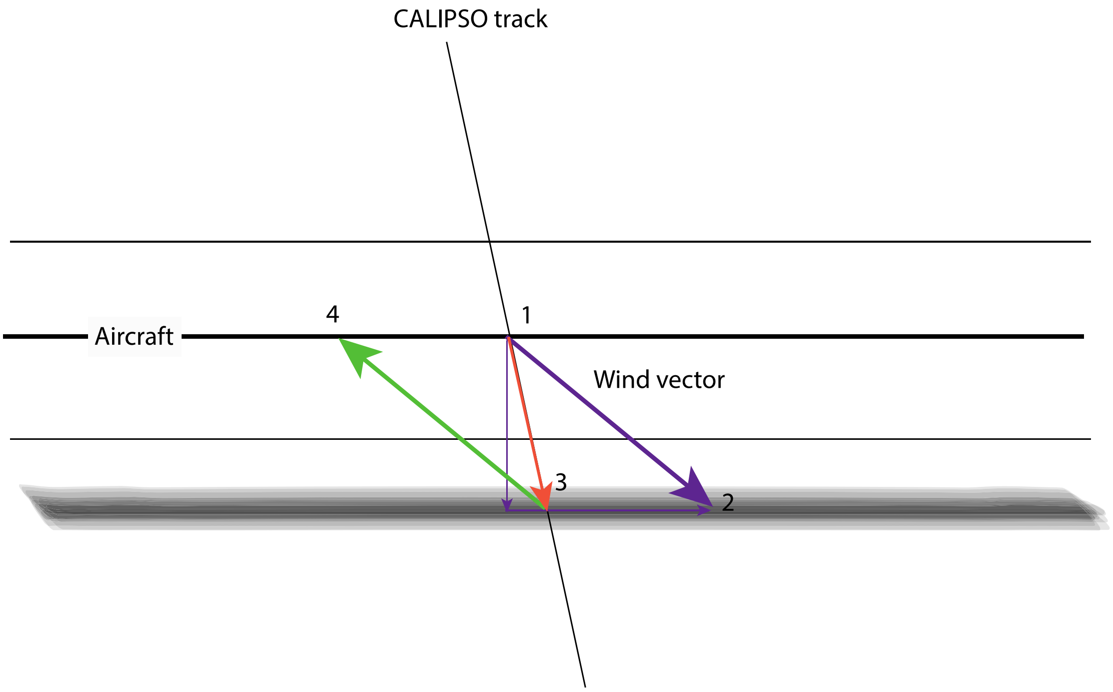

Supplementary Figure 3 | Example of advection geometry. The path of the aircraft is shown as a thick horizontal line. The Cloud-Aerosol Lidar and Infrared Pathfinder Satellite Observations (CALIPSO) satellite track is shown as a thin slanted vertical line. An arbitrary wind vector is shown as a thick purple arrow. The thin vertical and horizontal arrows depict the orthogonal components of the wind vector. The thin red arrow shows the component of the wind vector along the CALIPSO track. The green arrow indicates reverse advection from a point on the CALIPSO track to the source of the air observed there.

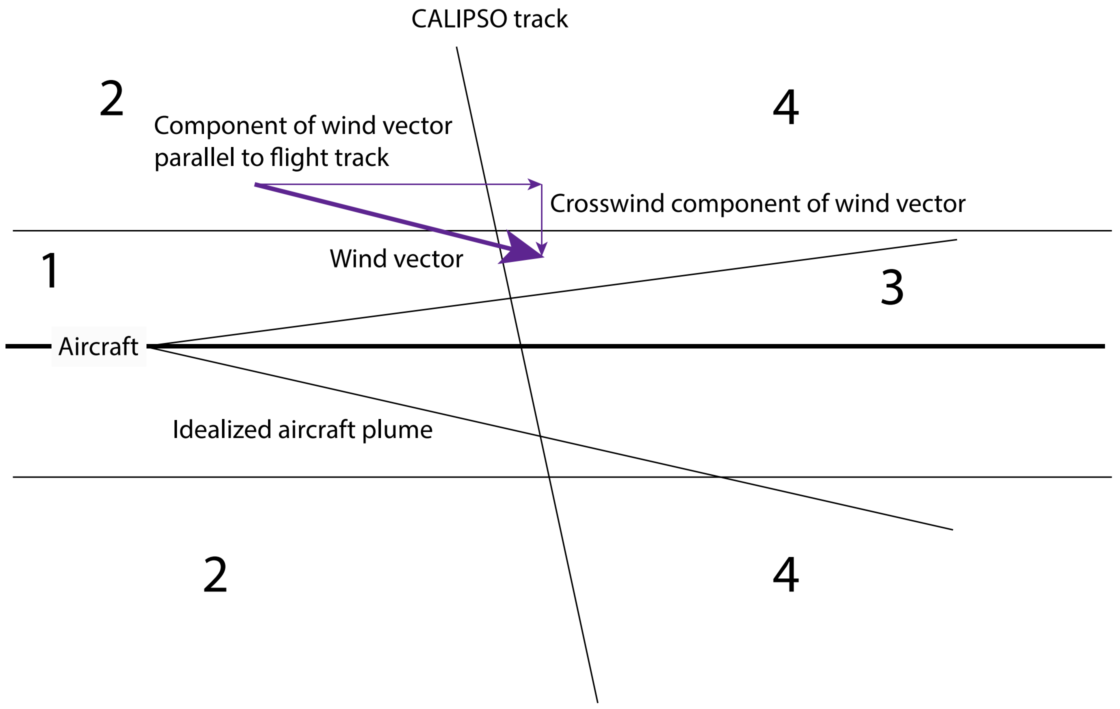

Supplementary Figure 4 | Potential effects of advection. The path of the aircraft is shown as a thick horizontal line. The Cloud-Aerosol Lidar and Infrared Pathfinder Satellite Observations (CALIPSO) satellite track is shown as a thin slanted vertical line. An arbitrary wind vector is shown as a thick purple arrow. The aircraft plume is depicted as a triangular area aft of the aircraft. The thin vertical and horizontal arrows depict the orthogonal components of the wind vector. The numbers indicate the four categories we use in our data analysis. The area along the plane track between the two thin horizontal lines is considered to be the flight corridor.

Supplementary Table 1 | Overview of the number of cases and data points per category suitable for analysis when accounting for displacement *D* perpendicular to the flight track and other aircraft on the same flight track with lead time *T*. The columns I/II and III/IV refer to CALIPSO passing the investigation area before the aircraft and vice versa.

	Number of cases			points per category			
Selection	All	I/II	III/IV	I	II	III	IV
all cases	109	50	59	240	262	340	354
D < 15 km	82	36	46	181	194	247	260
T > 30 min	99	46	53	222	235	310	329
D < 15 km, T > 30 min	69	29	40	142	154	219	227
D < 30 km, T > 30 min	91	44	47	215	225	276	288

Supplementary Table 2 | Overview of the number of data points, mean normalised cirrus optical thickness (nCOT), and p-values (Student's t test) for different combination in displacement *D* perpendicular to the flight track and lead time *T* of other aircraft on the same flight track for cirrus clouds with a geometrical depth below 2.5 km.

	points per category				*mean nCOT per category*				*p values*		
Selection	***I***	***II***	***III***	***IV***	***I***	***II***	***III***	***IV***	***III-I***	***III-II***	***III-IV***
all cases	*139*	*152*	*209*	*220*	*0.51*	*0.49*	*0.60*	*0.50*	*0.0016*	*0.0001*	*<0.0001*
D<15 km	*114*	*132*	*164*	*171*	*0.52*	*0.50*	*0.59*	*0.50*	*0.0560*	*0.0020*	*0.0022*
T>30 min	*129*	*130*	*189*	*198*	*0.50*	*0.48*	*0.61*	*0.49*	*0.0004*	*<0.0001*	*<0.0001*
D<15 km, T>30 min	*94*	*105*	*145*	*146*	*0.54*	*0.47*	*0.59*	*0.49*	*0.1530*	*0.0004*	*0.0019*
D<30 km, T>30 min	*122*	*122*	*173*	*181*	*0.50*	*0.47*	*0.60*	*0.48*	*0.0027*	*<0.0001*	*<0.0001*

Supplementary Discussion

The hypothesis we wish to test is "*contrails formed within natural cirrus clouds have no measurable immediate effect on cirrus cloud optical depth inside and outside flight tracks in the upper troposphere*."

If this hypothesis were false, then the optical thickness of clouds in sector III (inside the flight track, aft of the aircraft) would be different than in the other sectors.

We need to analyze whether there is any possibility that advection could move material emitted by the aircraft (or any other changes caused by the passage of the aircraft) between the regions we define in our analysis *in the time interval between the passage of the aircraft and the satellite overpass*. Supplementary Figure 4 illustrates the geometrical considerations for assessing the potential effects of advection.

Any case in which the dot product of the wind and aircraft velocity vectors is not identically 1 or -1 implies that there is a non-zero component of the wind vector orthogonal to the aircraft velocity vector – a crosswind. Formally, cases in which the dot product of the two vectors is quite close to 1 or -1 qualify as crosswinds yet still could serve to transport material between regions 1 and 3. For advection to move material from region 3 to region 1 sufficiently to influence a satellite observation made before the arrival of the aircraft would require that the tailwind velocity be (much) larger than the aircraft velocity – in which case the aircraft would not remain airborne for long. We discount this effect. A headwind could possibly dilute emissions in the flight corridor if the headwind velocity component was sufficiently large that it could transport material from another region to the point at which the satellite crosses the flight track in the time interval between when the aircraft and satellite pass this common point.
In reality the important advection effects –if they were important at all - would be for transport between regions I and II and regions III and IV.

In all these cases, advection would tend to reduce the difference in normalised cirrus optical thickness (nCOT) between sector III and the other sectors. If advection were a dominant effect, we would not be able to falsify our null hypothesis. We have shown in the manuscript (and in the additional material below) that we can clearly see an increase in nCOT in sector III compared to the other sectors. Thus the conclusion we can draw is that the effects of advection are not sufficiently strong to erase the influence of the aircraft on cloud properties.

We made no *a priori* assumptions about whether the effect of the aircraft would be to increase or decrease COT. Our assumption was that if the aircraft had an effect on already-existing clouds, region III would have *different* properties than the other categories. The data showed that this difference was an increase in COT, not a decrease. For purposes of analytical completeness, however, let us assume that the effect of the aircraft was to decrease COT, and that this effect

was advected away from region III into the downwind part of region IV as Reviewer 1 suggests. What would we observe?
First, we aggregate observations in region IV on both sides of region III. Since advection would only move the postulated region of decreased COT in the downwind direction, this would mean that any observations in this area of decreased COT would be combined with an equal number of observations in the other unaffected part of region IV. This would decrease the likelihood that we would observe a statistically significant difference in COT between regions III and IV, unless the decrease in COT was quite large. For the purposes of completeness, let us assume that this is indeed the case, and examine what the result would be.
In this case, the COT in region IV would be lower than that for regions I, II and III, and there would be no statistically significant differences in COT for these latter three regions. We do not observe this effect.

If the wind were roughly parallel to the flight track and in the same direction as the aircraft is traveling, it would tend to concentrate any material in the aircraft plume in sectors I and III relative to crosswind or headwind cases. This could possibly augment any differences between sectors I and III with sectors II and IV. Instances of aircraft heading-wind directions of 10° or represent about 20% of all cases (regardless of whether there are other aircraft in the vicinity); please see Figure S3 for a more detailed discussion of advection and winds. Since we still observe a statistically significant difference in nCOT between sectors I-III ($p=0.0016$) for all cases, this effect can be discounted.

In order to further assess potential effects of advection, we have stratified the data into five conditions of increasing restrictiveness:

1. All cases: All satellite/flight track crossings (109 cases);
2. T > 30 min: Satellite/flight track crossings were only included where the delay between any previous aircraft and our flight of interest was greater than 30 min (99 cases);
3. D < 30 km, T > 30 min: Satellite/flight track crossings where we limit the displacement of the data with respect to the centreline of the flight track to 30 km AND only include cases where the delay between any previous aircraft and our flight of interest was greater than 30 min (91 cases);
4. D < 15 km: Satellite/flight track crossings where we limit the displacement of the data with respect to the centreline of the flight track to 15 km (82 cases);
5. D < 15 km, T > 30 min: Satellite/flight track crossings where we limit the displacement of the data with respect to the centreline of the flight track to 15 km AND only include cases where the delay between any previous aircraft and our flight of interest was greater than 30 min (69 cases).

If advection were an important process diluting the effect of aircraft on cloud optical properties, and given a sufficient number of observations on which to

base statistical tests for differences in means, then we would expect the most restrictive case to produce the most statistically significant results.

Supplementary Table 1 gives the number of comparison cases and data points for each sector, while Supplementary Table 2 provides an overview of mean nCOT and p-values for sectors with statistically significant differences in nCOT. The tables show however that a more restrictive filtering leads to a strong decrease in the amount of data available for the analysis. Even so, Table 2 shows that for the cases where the strongest restriction has been applied (D <15 km and T< 30 min) statistically significant differences are found for sectors III-IV and III-II but not for the sectors III–I. Note however for the most relevant comparison (III-IV) we obtain a clear statistical significance ($p = 0.0019$) also with the most rigorous restriction. Differences in nCOT between the other sectors (e.g., I-II, I-IV) are not statistically significant.
